# CrusTome: a transcriptome database resource for large-scale analyses across Crustacea

**DOI:** 10.1093/g3journal/jkad098

**Published:** 2023-05-02

**Authors:** Jorge L Pérez-Moreno, Mihika T Kozma, Danielle M DeLeo, Heather D Bracken-Grissom, David S Durica, Donald L Mykles

**Affiliations:** Department of Biology, Colorado State University, Fort Collins, CO 80523, USA; Department of Biology, Colorado State University, Fort Collins, CO 80523, USA; Department of Invertebrate Zoology, National Museum of Natural History, Smithsonian Institution, Washington, DC 20560, USA; Department of Invertebrate Zoology, National Museum of Natural History, Smithsonian Institution, Washington, DC 20560, USA; Department of Biological Sciences and Institute of Environment, Florida International University, North Miami, FL 33181, USA; Department of Biology, University of Oklahoma, Norman, OK 73019, USA; Department of Biology, Colorado State University, Fort Collins, CO 80523, USA

**Keywords:** Arthropoda, bioinformatics, BLAST, crustaceans, cryptochrome, phylogenetics, RNA-seq

## Abstract

Transcriptomes from nontraditional model organisms often harbor a wealth of unexplored data. Examining these data sets can lead to clarity and novel insights in traditional systems, as well as to discoveries across a multitude of fields. Despite significant advances in DNA sequencing technologies and in their adoption, access to genomic and transcriptomic resources for nontraditional model organisms remains limited. Crustaceans, for example, being among the most numerous, diverse, and widely distributed taxa on the planet, often serve as excellent systems to address ecological, evolutionary, and organismal questions. While they are ubiquitously present across environments, and of economic and food security importance, they remain severely underrepresented in publicly available sequence databases. Here, we present CrusTome, a multispecies, multitissue, transcriptome database of 201 assembled mRNA transcriptomes (189 crustaceans, 30 of which were previously unpublished, and 12 ecdysozoans for phylogenetic context) as an evolving and publicly available resource. This database is suitable for evolutionary, ecological, and functional studies that employ genomic/transcriptomic techniques and data sets. CrusTome is presented in BLAST and DIAMOND formats, providing robust data sets for sequence similarity searches, orthology assignments, phylogenetic inference, etc. and thus allowing for straightforward incorporation into existing custom pipelines for high-throughput analyses. In addition, to illustrate the use and potential of CrusTome, we conducted phylogenetic analyses elucidating the identity and evolution of the cryptochrome/photolyase family of proteins across crustaceans.

## Introduction

A distinct paucity of readily available genomic and transcriptomic resources persists for nonmodel organisms, despite recent advances in sequencing technologies and adoption of bioinformatics across diverse fields (Mykles *et al.* 2016; Burnett *et al.* 2020). Nonmodel organisms often harbor a wealth of useful genomic and transcriptomic data, which can lead to discoveries and unforeseen advances in a diverse array of seemingly unrelated areas (GIGA Community of Scientists 2014; Tagu *et al.* 2014). Crustaceans are among the most numerous and diverse taxa on the planet (Martin and Davis 2006; Ahyong *et al.* 2011; Schram 2013). Thanks to their ubiquitous presence across an extreme diversity of biomes (Pérez-Moreno *et al.* 2016; Bracken-Grissom and Wolfe 2020), they are particularly well suited to address questions of ecological, evolutionary, and organismal interest (Stillman *et al.* 2008; Pérez-Moreno *et al.* 2018; Wolfe *et al.* 2021). In addition to their critical environmental and scientific relevance, crustaceans are of major significance for social, economic, and food security implications (Timm *et al.* 2019; Boyd *et al.* 2022). Nevertheless, similar to other nonmodel invertebrates, crustaceans are severely underrepresented in publicly accessible (and readily available) databases such as those maintained by the National Center for Biotechnology Information (NCBI) (GIGA Community of Scientists 2014; Havird and Santos 2016; Hyde *et al.* 2020). Obtaining data from raw read databases, such as the NCBI Sequence Read Archive (SRA), and transforming them into a useable format represents a time-consuming and computationally expensive process. The ability to search the Transcriptome Shotgun Assembly (TSA) database and extract data in a high-throughput manner is challenging, as most TSA transcriptomes are assembled through different methodologies. These limitations ultimately hinder accessibility of crustacean transcriptomes for use by nonspecialists and researchers with limited computational, temporal, or financial resources.

Previous efforts in developing resources utilizing crustacean transcriptomic data include CrustyBase, CrusTF, and Crustacean Annotated Transcriptome (CAT) databases among others (Qin *et al.* 2017; Nong *et al.* 2020; Hyde *et al.* 2020). CrustyBase provides access to transcriptomes from 17 crustacean species through a graphical interface for BLAST searches and evaluation of gene expression. Assembled transcriptomes and expression data in CrustyBase are uploaded by individual researchers or research groups. CrusTF is available as a graphical interface database dedicated to capture transcription factors detected in transcriptomes of 170 crustacean species. The CAT database is an annotated resource of multiple transcriptomes generated from 7 crustacean species that is accessible for BLAST searches via a web-based graphical interface.

Here, we present CrusTome (a portmanteau from Crustacea and the Greek word *tomos*, book or volume): a multispecies and multitissue database of assembled mRNA transcriptomes from 201 species (currently, 189 crustaceans across 16 orders, 30 species which were previously unpublished, and 12 additional representatives from among Ecdysozoa). The goal of developing CrusTome is to aid in evolutionary, ecological, and functional studies that employ genomic/transcriptomic techniques for sequence similarity searches, orthology assignments, and phylogenetic inference, among other uses. CrusTome was generated by assembling transcriptomic raw reads available in public repositories from a variety of tissues of individual crustacean species, along with previously unpublished transcriptomic raw reads. Transcriptomes of each species were assembled utilizing similar methodology (discussed below) and processed to remove microbial contamination and redundancy. By incorporating the evidential gene pipeline (Gilbert 2019), a single refined transcriptome was generated for each species and tissue type. These refined transcriptomes were then combined to form a single database, encompassing all selected species, that is presented in BLAST and DIAMOND formats of assembled contigs and their predicted peptides. Presenting the database in these 2 formats allows for simple and straightforward incorporation into existing custom analysis pipelines, making it particularly suitable for scripting and high-throughput analyses (e.g. Pérez-Moreno *et al.* 2018; Drozdova *et al.* 2021). The database will be updated regularly by assembling and incorporating raw reads generated from new species and tissues that are made available in public repositories. Additionally, when advances in assembly software or bioinformatic pipelines warrant the reprocessing or reassembly of the raw reads, updated versions will be released. The development of CrusTome was made possible using a high-memory computing node at the Supercomputing Center for Education & Research (OSCER) at the University of Oklahoma.

To showcase the utility and power of CrusTome, we present here an example in which we conducted the first large-scale transcriptomic exploration across crustaceans of the cryptochrome/photolyase family (CPF). Cryptochromes and photolyases are UV-A/blue-light sensitive proteins that can be found across the entire tree of life and share a common general structure of a conserved photosensory domain bound to 2 chromophore cofactors (Sancar 2003, 2008; Chaves *et al.* 2011; Oliveri *et al.* 2014; Mei and Dvornyk 2015). They are light-sensitive flavoproteins involved in DNA repair, circadian rhythm regulation, and magnetoreception that have also shown promising applications as optogenetic tools (Oliveri *et al.* 2014; Mei and Dvornyk 2015; Hernández-Candia and Tucker 2020; Kiontke *et al.* 2020). Despite their ubiquity and functional diversification, little is known about CPFs in crustaceans, and, as such, they present an ideal opportunity to illustrate CrusTome's potential for phylogenetic characterization. The code and scripts used to generate this analysis are made accessible, providing readers with a highly customizable framework and pipeline that begin with BLAST searches across CrusTome and end in developing highly refined phylogenies of gene families. By utilizing CrusTome in combination with a phylogenetic pipeline, carcinologists can better annotate transcriptomes using a phylogenetically informed evolutionary perspective. With the computational resources available to us through OSCER, we aim to aid fellow researchers in assembling and incorporating their crustacean transcriptomic data sets into CrusTome, thereby crowdsourcing an improved taxonomic representation of crustaceans in the -omics era.

Links to download the CrusTome database, associated metadata, future updates, and the code to reproduce the example analysis herein are available at CrusTome's GitHub site: https://github.com/invertome/crustome. Direct links to CrusTome v0.1.0 BLAST and DIAMOND formats are presented under the *Data availability* section of this manuscript.

## Methods

### Data sourcing

**Fig. 1.**
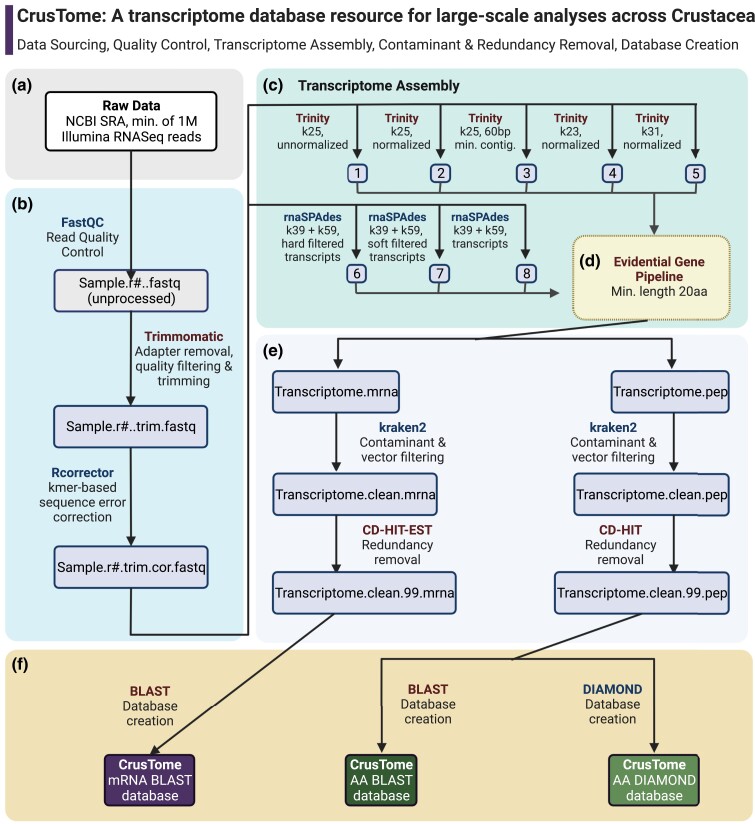
Flowchart of the CrusTome database creation steps. a) Data sourcing. b) Quality control and preprocessing. c) Transcriptome assembly with multiple assemblers. d) Obtaining optimal transcriptome via EVG pipeline. e) Contamination and redundancy filtering. f) Creation of BLAST and DIAMOND databases. Figure created with Biorender.com.

Transcriptomes were assembled from raw RNA-sequencing (RNA-seq) reads that were publicly available. RNA-seq reads from 30 species that were previously unpublished are also included. Our usage of “raw reads” refers to RNA-seq reads exclusively. Emphasis was placed on raw reads of nonhexapod *Pancrustacea* samples (*n* = 189) covering the phylogenetic breadth available on the NCBI SRA database (excepting *Hexapoda*; Leinonen *et al.* 2011), with representation of 16 crustacean orders. Our usage of “crustacean(s)” refers to nonhexapod pancrustaceans. Criteria for selecting raw reads included (1) the use of next-generation sequencing (NGS) technologies to generate raw reads and (2) a minimum read depth of 1 M reads per sample downloaded (Fig. 1a). Both criteria allowed for the assembly of complete and contiguous transcriptomes, while avoiding fragmentation issues due to low sequencing depth. Raw reads generated from 12 ecdysozoan and hexapod species were included to serve as outgroups and to provide phylogenetic context, respectively, during gene characterization and phylogenetic inference. Raw reads of nematodes from the family *Monhysteridae* were included as an outgroup and to assist in identifying possible contaminant sources. Species from the *Monhysteridae* family frequently occur as endoparasites or in association with numerous crustacean species (Baylis 1915; Chitwood 1935; Tchesunov and Ivanenko 2022; Westerman *et al.* 2022) and were thus deemed useful as a mechanism to filter out potential nonarthropod contaminant sequences during downstream analyses. Full details on the species and their tissues included, accession identifiers, and corresponding raw read and transcriptome metadata can be found in Supplementary File 1.

### Quality control

Raw reads downloaded from the NCBI SRA database were visually inspected using FastQC (Andrews 2010) to determine read filtering, trimming stringency, and thresholds to be applied across samples in a standardized manner. As samples were of a heterogeneous nature with a range of input qualities, trimming settings were set up conservatively to ensure proper transcriptome assemblies and the reliability of downstream analyses by avoiding fragmentation issues due to incomplete removal of index/barcode sequences and erroneous base-calls by the sequencing instrument. Automated trimming of the sequencing reads was undertaken using Trimmomatic (Bolger *et al.* 2014) with the following settings: *CROP*=“*x*” *HEADCROP*=“*15*” *MINLEN*=“*45*” *SLIDINGWINDOW*=“*4:20*” *LEADING*=“*15*” *TRAILING*=“*15*”, with the *CROP* value “*x*” adjusted to remove the error-prone final 10 error-prone bases according to each sequencing library's fragment length (e.g. 90 for 100 bp reads or 140 for 150 bp reads). Subsequent to trimming, the resulting reads were piped into Rcorrector using default settings (Song and Florea 2015). *k*-mer–based random sequencing error correction of Illumina reads used a De Bruijn graph algorithm, which is particularly suitable for error correction of RNA-seq reads (Song and Florea 2015; MacManes 2018; Ortiz *et al.* 2021; Fig. 1b).

### Transcriptome assembly

The quality-filtered, trimmed, and error-corrected reads were assembled into de novo transcriptomes for each sample using a multiassembler approach (Fig. 1c), which leverages the advantages of different assembly algorithms and parameters to obtain a single optimal, and less fragmented, transcriptome assembly (Nakasugi *et al.* 2014; MacManes 2018; Gilbert 2019; Ortiz *et al.* 2021). For each species, 8 transcriptomes were initially assembled via several iterations of the Trinity pipeline (version 2.13.2; Grabherr *et al.* 2011; Haas *et al.* 2013) and rnaSPAdes (Bankevich *et al.* 2012). For each Trinity assembly, different parameters were specified for *k*-mer, normalization, and minimum contig length (*k25* + *unnormalized reads*, *k25* + *normalized reads*, *k25* + *60 bp min contig length*, *k31* + *normalized*, and *k23* + *normalized*). Only those contigs that were supported by read mappings were retained (Haas *et al.* 2013). Additional assemblies were produced using rnaSPAdes (version 3.15.13; Bankevich *et al.* 2012) and its integrated multi–*k*-mer assembly approach. rnaSPAdes was run with 2 *k*-mer settings (*k*39 + *k*59) after which assembled contigs were collated into 3 assemblies resulting from differing quality filtering thresholds (*hard_filtered_transcripts.fasta*, *soft_filtered_transcripts.fasta*, and *transcripts.fasta*), all of which were included in subsequent merging steps. All of the Trinity and rnaSPAdes assemblies (*n* = 8) for each sample were then merged into a single optimal transcriptome via the EvidentialGene (EVG) pipeline (Gilbert 2019), with a selected minimum amino acid length of 20 residues (e.g. to allow for the detection and characterization of small neuropeptides; Fig. 1d). This multiassembler and multiparameter design produces transcriptome assemblies of higher quality and completeness (MacManes 2018; Gilbert 2019; Ortiz *et al.* 2021), which are illustrated by summary statistics and Benchmarking Universal Single-Copy Orthologs (BUSCO) scores (Simão *et al.* 2015) among other metrics of protein completeness (Gilbert 2019). The final merged transcriptomes were filtered with the EVG pipeline for long noncoding RNA (lncRNA) sequences, which are beyond CrusTome's current scope, to improve the efficiency of sequence similarity searches and downstream analyses. The filtered mRNA transcriptomes were then translated into amino acid sequences to produce both transcribed and translated versions for convenience and accessibility. Samples from previously assembled data (namely *Daphnia* and hexapods) were included in their original TSA versions and processed through the EVG pipeline for consistency and suitability of comparisons. It is important to note that included samples continue to be subject of reassembly and will be included in their final multiassembler versions in upcoming releases of CrusTome. Transcriptome assembly and associated bioinformatic analyses were performed using computational resources provided by the Instructional & Research Computing Center (IRCC) at Florida International University and the OSCER at University of Oklahoma.

### Contaminant filtering and redundancy removal

Both mRNA and amino acid transcriptomes for each sample were subsequently filtered for contamination using Kraken 2.1.2 (Wood *et al.* 2019; Wright *et al.* 2022) and a custom database that included archaea, bacteria, virus, fungi, and sequencing vector sequences (obtained from https://lomanlab.github.io/mockcommunity/mc_databases.html), as well as mouse and human sequences (Bushnell 2018; to remove possible contamination arising from sequencing facilities), with settings optimized for filtering crustacean transcriptomes (“--*confidence 0.1*”; Wright *et al.* 2022). It is important to note that the confidence setting employed was determined in an iterative process following Wright *et al.* (2022) to filter out most contaminant sequences with minimal loss of crustacean sequences. However, it is possible that some noncrustacean sequences from symbionts may still be present in the database: further filtering steps according to each specific scenario are highly encouraged (e.g. via a phylogenetic assessment). A final application of the CD-HIT-EST and CD-HIT (“Cluster Database at High Identity with Tolerance”) clustering algorithm was run on each individual EVG-optimized transcriptome (mRNA and amino acid, respectively) to cluster contigs at a 99% sequence identity (Fig. 1e). This allowed for the removal of contigs likely to have been produced from sequencing errors, while minimizing the removal of true isoforms.

### Transcriptome summary statistics and completement assessment

TransRate version 1.0.3 and BUSCO version 3.0.2 were used to calculate summary statistics and to assess the completeness of the CrusTome transcriptome assemblies (Simão *et al.* 2015; Smith-Unna *et al.* 2016) (Supplementary File 1). BUSCO analyses were conducted using OrthoDB's Arthropoda database of orthologous groups (Waterhouse *et al.* 2013) as a reference data set (OrthoDB v10).

### BLAST and DIAMOND database creation

The CrusTome transcriptome and predicted amino acid databases were created in 2 formats using default settings, as both BLAST and DIAMOND databases (Altschul *et al.* 1990; Buchfink *et al.* 2021; Fig. 1f) for compatibility with annotation and analysis pipelines (e.g. see Das *et al.* 2016; Pérez-Moreno *et al.* 2018; Tang *et al.* 2019; Drozdova *et al.* 2021). DIAMOND is an ultrafast alignment software that achieves considerable sequence similarity search speeds by orders of magnitude faster than BLAST, at a minimum sensitivity cost (Buchfink *et al.* 2021), and, as such, is appropriate for the high-throughput applications now available with CrusTome.

### “CrusTome” example pipeline

An example analysis was conducted to illustrate the potential of CrusTome for the identification and characterization of proteins of interest. Specifically, we conducted a series of recursive BLAST searches against CrusTome's predicted amino acid sequence database, followed by an alignment and phylogenetic inference strategy to gain insight into the presence and expression of DNA–photolyases, cryptochromes, and “*Drosophila*, *Arabidopsis*, *Synechocystis*, and Human” (DASH)-like cryptochromes (Oliveri *et al.* 2014; Mei and Dvornyk 2015; Kiontke *et al.* 2020) across crustaceans.

The phylogenetically informed annotation analyses consisted of an initial BLAST search against CrusTome using reference cryptochrome and DNA–photolyase sequences previously characterized in insects (Supplementary File 2), specifying a maximum number of hits of 500 to capture as much sequence diversity as possible (Shah *et al.* 2019), but with a relatively stringent *e*-value of *e*^−120^ to limit results to relevant peptides. The list of hit IDs resulting from this initial search was then used to extract the corresponding sequences from CrusTome, which were then used as input for a second BLAST search against the database to capture additional sequence diversity. Sequences identified as hits from this second BLAST iteration were once again extracted from CrusTome. These putative peptides identified by BLAST were subsequently concatenated with the insect references originally employed as search queries, which were then aligned with the multiple sequence aligner Multiple Alignment using Fast Fourier Transform (MAFFT, v.7.490; Yamada *et al.* 2016). The MAFFT software was used to align putative cryptochrome and photolyase sequences obtained from the CrusTome database, along with the original insect reference sequences used as BLAST queries. MAFFT alignment parameters were specifically chosen to prioritize accuracy over speed and to allow for large unalignable regions that can be pervasive in certain protein families (“--*dash* --*ep 0* --*genafpair* --*maxiterate 1000*”; see Yamada *et al.* 2016). The --*dash* parameter enables MAFFT to query a Database of Aligned Structural Homologs, providing structural information with which to refine the alignment process (Rozewicki *et al.* 2019). The resulting alignment was then trimmed using ClipKit (smart-gap mode) (Steenwyk *et al.* 2020), which identifies and retains phylogenetically informative sites for a more accurate and robust phylogenetic inference. Maximum likelihood phylogenetic reconstruction was undertaken with IQ-TREE2 (Nguyen *et al.* 2015) with a Le–Gascuel (LG) general amino acid replacement matrix under a FreeRate model with 10 rate categories (LG + R10; Yang 1995; Müller and Vingron 2000; Le and Gascuel 2008; Soubrier *et al.* 2012), as recommended for the trimmed alignment by ModelFinder (Kalyaanamoorthy *et al.* 2017). The phylogenetic tree resulting from this initial reconstruction was then piped, in conjunction with the alignment, to TreeShrink for outlier/paralog detection and removal at an *α*-value of 0.05 (Mai and Mirarab 2018). The resulting pruned alignment was then used for a second and final phylogenetic reconstruction with IQ-TREE2 (Nguyen *et al.* 2015) for characterization and annotation of the putative peptides. A second IQ-TREE2 phylogenetic reconstruction was run using the same model parameters previously reported (LG + R10; Yang 1995; Müller and Vingron 2000; Le and Gascuel 2008; Soubrier *et al.* 2012). Branch support of this final phylogeny was assessed in bipartite by Ultra-Fast Bootstrap approximation (UFBoot; 10,000 replicates) and an approximate Bayes test (Guindon *et al.* 2010; Anisimova *et al.* 2011; Minh *et al.* 2013). Finally, the resulting phylogenies were used to classify the obtained peptide sequences as members of cryptochrome 1, cryptochrome 2, DASH-like cryptochromes, 6-4 pyrimidine-pyrimidone photoproduct (6-4) photolyases, or cyclobutane pyrimidine dimer (CPD) photolyases as per previous studies in other organisms (Oliveri *et al.* 2014; Mei and Dvornyk 2015; Kiontke *et al.* 2020). Protein sequences were collated for each of these major clades, and each of the sequence groups was then aligned following the previously mentioned strategy. The gene-specific alignments were then used to generate hidden Markov model profiles with HMMER (Finn *et al.* 2011; Eddy 2011). These profiles are made available with CrusTome as a community resource. Example code for this phylogenetic analysis is included as Supplementary File 3.

## Results and discussion

The underrepresentation of nonhexapod pancrustaceans in publicly available databases is largely attributed to challenges of a technical nature, rather than to a lack of effort or adoption of genomic methodologies by researchers. This disparity is exemplified by the rapid increase of raw sequencing reads in the NCBI SRA (Havird and Santos 2016; Qin *et al.* 2017; Hyde *et al.* 2020), in contrast to the TSA database. The TSA database contains transcriptome assemblies that are submitted to GenBank from the research community. Consequently, these assemblies are highly heterogeneous, in terms of sequencing and/or assembly methods, fragmentation, redundancy, quality, metadata content, and annotation. For these reasons, many studies producing large amounts of transcriptomic data now opt to submit raw sequencing files to public repositories. As the SRA database is composed of raw sequencing reads, accessing information stored therein requires a specific expertise and set of skills, oftentimes with steep learning curves. Given that crustacean “omics” data are now being produced at a far greater rate than can be meaningfully accessed, analyzed, and interpreted by many researchers, CrusTome delivers a solution that is simple to implement to enable large-scale transcriptomic analyses across nonhexapod *Pancrustacea*. The multiple *k*-mer assembly strategy and subsequent merging through the EVG pipeline used for CrusTome offers noticeable advantages for de novo transcriptome assembly from organisms without a reference genome (Gilbert 2019; Summary Statistics & BUSCO Assessment, Supplementary File 1). Additionally, special emphasis was placed on the processing of the publicly available data by reassembling and processing each included transcriptome with the consistent pipeline (Fig. 1), rather than including assemblies produced by disparate methodologies. This uniform processing provides a standardization for accurate downstream analyses.

Several approaches have been used previously to address this knowledge gap for representing crustaceans and other nontraditional model organisms (Qin *et al.* 2017; Nong *et al.* 2020; Hyde *et al.* 2020). For example, CrusTF is a web-based database resource containing sequence data, with an emphasis on transcription factors, mined from multiple transcriptomic sources (Qin *et al.* 2017). Having sourced data from over 170 transcriptomes, CrusTF is the most taxonomically diverse curated database available to date. However, despite the taxonomic breadth covered across *Crustacea*, ease of access, and web-based tools and operability, its scope is limited, as it pertains exclusively to transcription factors and is only accessible through a graphical interface. The CAT database consists of a web-based interface to conduct BLAST searches against 71 transcriptomes, but only across 7 species (Nong *et al.* 2020). Moreover, as a web interface–based database, CAT is not available for high-throughput analyses, scripting, or incorporation into bioinformatic pipelines. One difference is that CAT is an annotated database, and annotations are not currently within the scope of CrusTome's current version. This is mainly due to the difficulty for providing accurate annotations for nonmodel crustaceans using sequence similarity search-based software and relying on curated databases more apt for common model organisms. CrustyBase, a recently published interactive database of crustacean transcriptomes, also employs a web-based approach with a GUI that excels in terms of accessibility, navigation, and operability (Hyde *et al*. 2020). It also leverages the advantages of being able to process gene expression data that can be linked directly to each submitted transcriptome, an integrated BLAST interface, and intuitive visualization features. Nevertheless, although highly curated, it is dependent upon direct submissions from the community and suffers from underrepresentation of numerous crustacean taxa. Furthermore, similar to CrusTF, CrustyBase's main target audience consists of those comfortable with conducting analyses exclusively through GUIs. While the GUI presentation is of great advantage for data accessibility to a specific sector that may be unfamiliar with coding, the utilization of these databases by those who wish to incorporate their data sets into existing command line–based bioinformatic pipelines for large-scale and/or high-throughput analyses is limited. CrusTome bridges this gap by providing the entire database in downloadable formats.

**Fig. 2.**
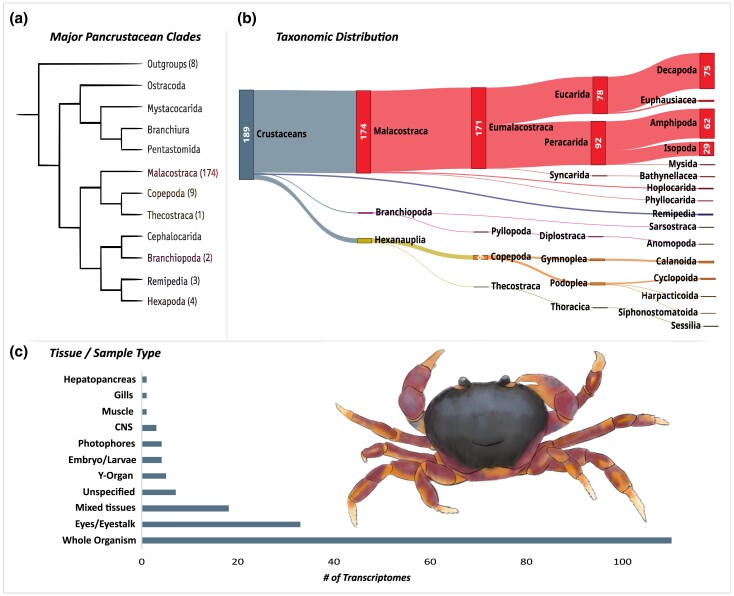
a) Taxon coverage across major pancrustacean clades in the current version of the CrusTome database. Phylogeny adapted from Oakley *et al.* (2013) and Bracken-Grissom and Wolfe (2020). b) Sankey diagram depicting the taxonomic distribution of transcriptomes included in the present version of the database. c) Tissue type distribution of CrusTome transcriptomes across nonhexapod pancrustaceans. Illustration of *Gecarcinus lateralis* by An-Ping Yu.

CrusTome's current version consists of a multispecies and multitissue transcriptome database from 189 nonhexapod pancrustacean species, including 30 previously unpublished transcriptomes and 12 additional ecdysozoan species (see Supplementary File 1 for additional details). This initial version of CrusTome includes a sample of resources currently available on the NCBI's public repositories and therefore is subject to similar representation biases (Fig. 2a). Consequently, CrusTome should be considered an evolving database resource, as it will continue to be updated to bridge these gaps whenever relevant data become available. The present version presents an uneven distribution of samples across pancrustacean taxa biased towards the class *Malacostraca*, which comprises 174 out of 201 transcriptomes (Fig. 2b). Despite this apparent overrepresentation, CrusTome includes representatives of rare and obscure taxa that present intriguing opportunities for phylogenetics, systematics, and evolution, such as remipedes and bathynellaceans (Pérez-Moreno *et al.* 2016) and multiple deep-water malacostracans (DeLeo and Bracken-Grissom 2020; Drozdova *et al.* 2021). Samples of pancrustacean taxa *Ostracoda*, *Mystacocarida*, *Branchiura*, and *Cephalocarida* are currently in the processing queue for upcoming iterations, improving CrusTome's phylogenetic breadth. In addition, other members of *Pancrustacea* (namely hexapods), as well as a select number of the subphylum *Chelicerata* and phylum *Tardigrada* transcriptomes, have been included to aid in comparative analysis and serve as outgroups to root phylogenies, along with nematodes from the family *Monhysteridae* to filter potential contamination from symbiotic organisms and/or parasites (Baylis 1915; Westerman *et al.* 2022). In addition to phylogenetic diversity, CrusTome also provides a wide array of sample types, from single tissues to whole organisms, aiming to encompass transcript diversity both across and within species (Fig. 2).

### Example analysis: large-scale exploration of cryptochromes and DNA–photolyases across Crustacea

To illustrate the functionality of the CrusTome database, an analysis was conducted to annotate previously uncharacterized CPF proteins expressed across multiple tissues from species spanning the pancrustacean phylogenetic tree. Cryptochromes and photolyases are found across the entire tree of life that share a common general structure, consisting of a conserved photosensory domain bound to 2 chromophore cofactors (Sancar 2003; Chaves *et al.* 2011; Oliveri *et al.* 2014; Mei and Dvornyk 2015). However, important functional differences exist between the 2 types of CPF proteins. Photolyases are light-dependent DNA repair enzymes that can be classified based on the type of damage they repair: (1) the CPD photolyases and (2) the 6-4 photolyase (Sancar 2003, 2008; Hitomi *et al.* 2009; Oliveri *et al.* 2014). Despite their structural similarity with photolyases, cryptochromes are not involved in DNA repair activity and instead participate in a wide variety of functions, such as light perception, transcriptional regulation, and magnetoreception (Chaves *et al.* 2011; Liu *et al.* 2011; Oliveri *et al.* 2014; Bazalova *et al.* 2016). Although CPF proteins are known to be present in all types of organisms (prokaryotic and eukaryotic; Reitzel *et al.* 2010; Rivera *et al.* 2012; Zantke *et al.* 2013), including crustaceans (i.e. the isopod *Eurydice pulchra* and the Antarctic krill *Euphausia superba*; Teschke *et al.* 2011; Zhang *et al.* 2013), little is known about their distribution and function across *Pancrustacea*.

**Fig. 3.**
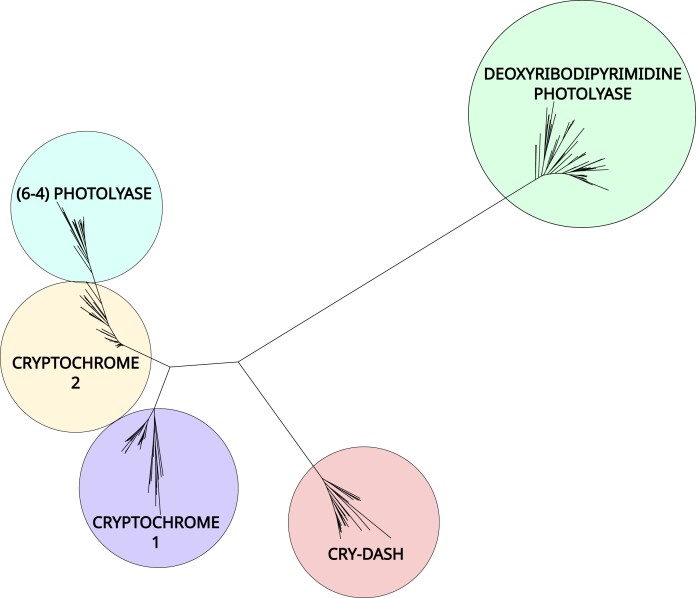
Unrooted phylogenetic tree (LG + R10) of cryptochrome 1, cryptochrome 2, cryptochrome-DASH, and photolyases in crustacean transcriptomes within the CrusTome database.

**Fig. 4.**
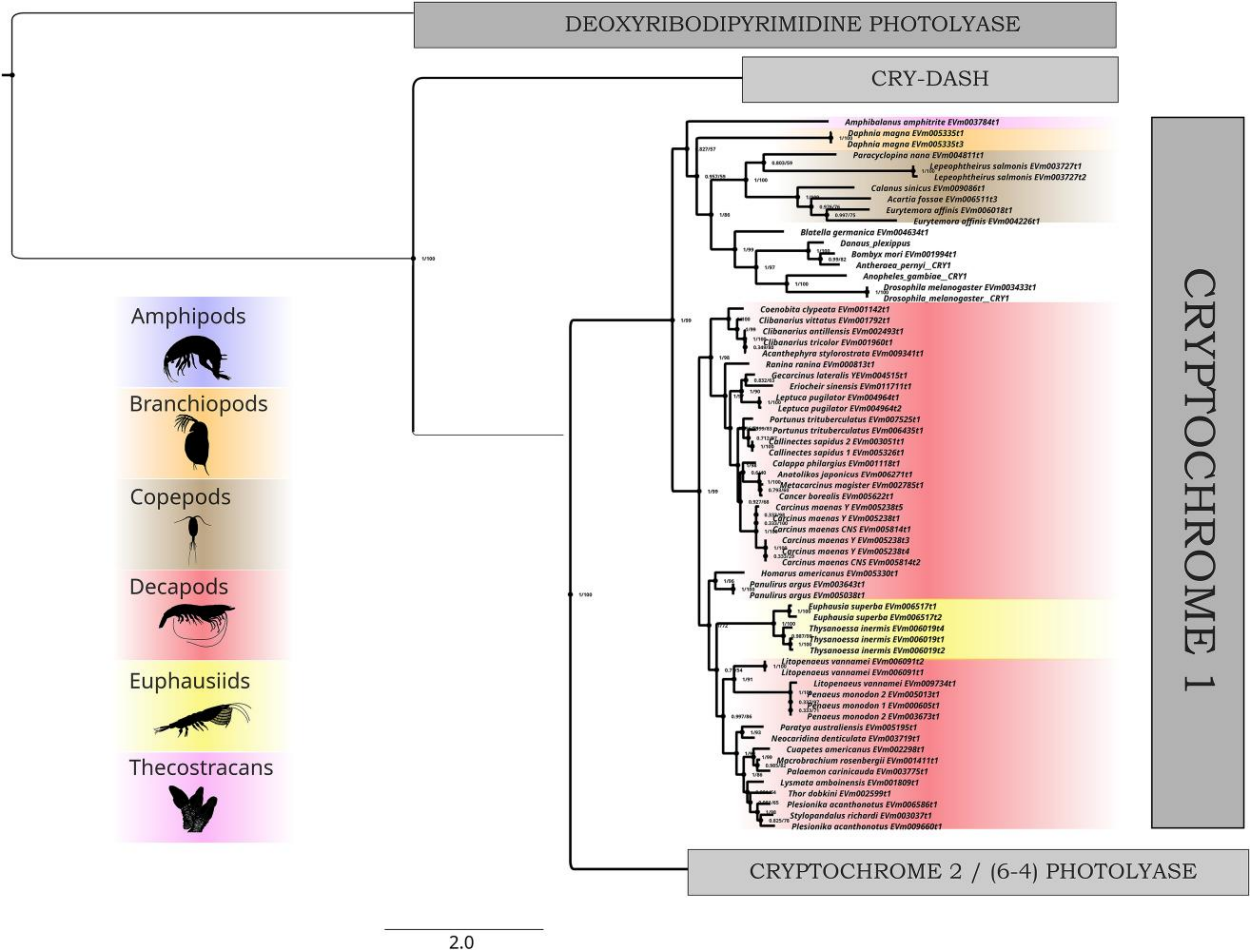
Rooted phylogenetic tree (LG + R10) of cryptochrome 1 found across transcriptomes of multiple crustacean species and tissues within the CrusTome database. Representative taxa images from PhyloPic.org: Amphipoda and Decapoda (Christoph Schomburg), Branchiopoda (T. Michael Keesey), Copepoda and Thecostraca (Joanna Wolfe), and Euphausiidae (Steven Haddock).

**Fig. 5.**
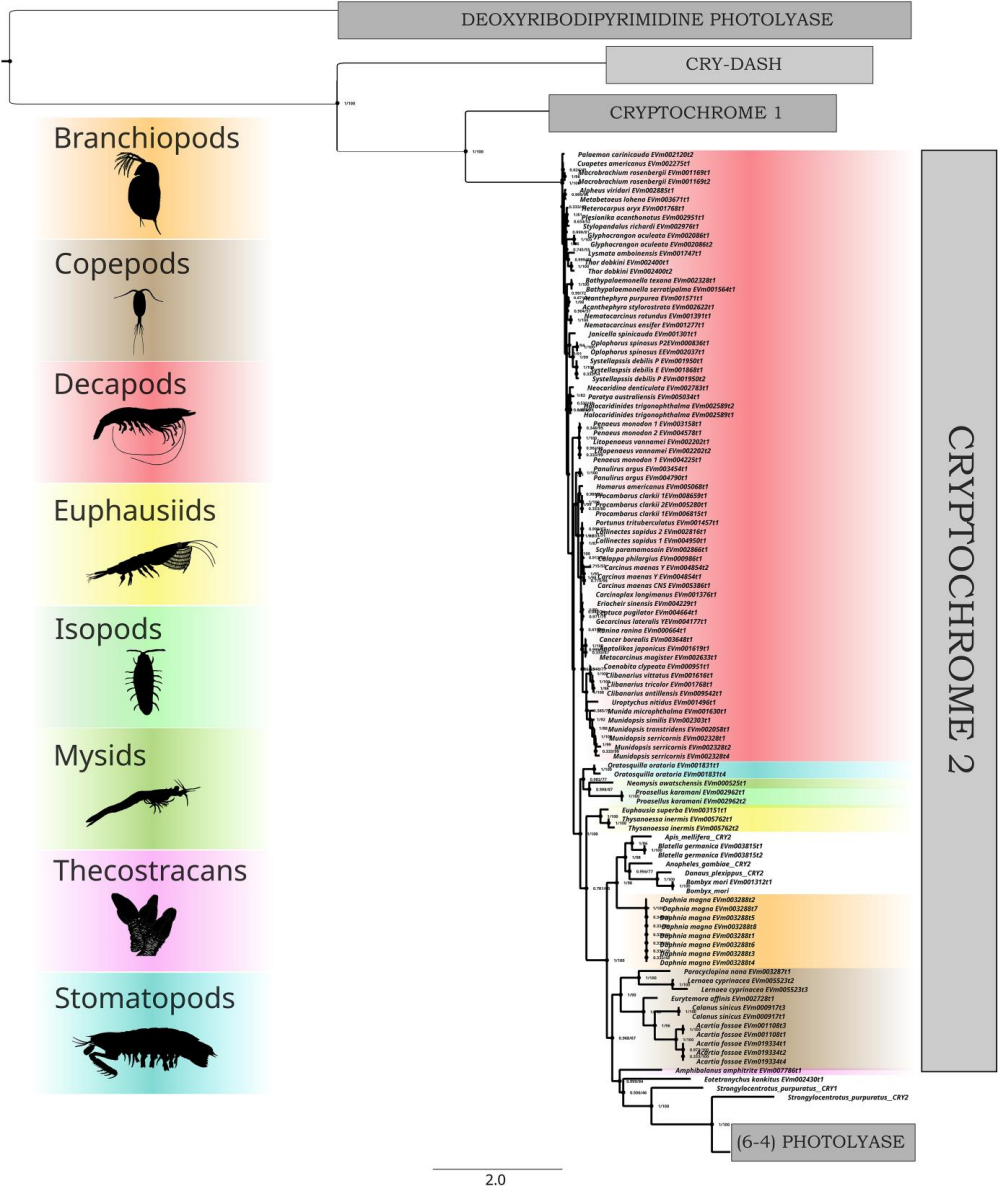
Rooted phylogenetic tree (LG + R10) of cryptochrome 2 found across transcriptomes of multiple crustacean species and tissues within the CrusTome database. Representative taxa images from PhyloPic.org: Branchiopoda and Stomatopoda (T. Michael Keesey), Copepoda and Thecostraca (Joanna Wolfe), Decapoda (Christoph Schomburg), Euphausiidae (Steven Haddock), Isopoda (Kanchi Nanjo), and Mysida (Denis Lafage).

Sequence similarity searches with BLAST (Altschul *et al.* 1990), using reference CPF sequences from the NCBI GenBank (sequences and accession IDs in Supplementary File 1), recovered putative CPF proteins from CrusTome's amino acid sequence database. A total of 382 unique sequences were obtained from the 201 transcriptomes included in the database. These sequences were subsequently aligned and trimmed, then used for phylogenetic reconstruction. The phylogram represented 5 major CPF clades, which corresponded to CPD photolyases, cryptochrome 1, cryptochrome 2, CRY-DASH, and 6-4 photolyases (Fig. 3), whose phylogeny was in overall agreement with previous work (Lin and Todo 2005; Lucas-Lledó and Lynch 2009; Mei and Dvornyk 2015). All of these groups formed monophyletic clades, with the exception of the 6-4 photolyases, which interestingly fell in their entirety as a clade within Cry2 sequences (additional phylograms in Supplementary File 4). This is consistent with a CPF phylogenetic analysis that found 6-4 photolyase and cryptochrome sequences cluster together, in contrast with other homologs (Mei and Dvornyk 2015). Differences in the taxonomic distribution of the 5 major clades are immediately evident, particularly between the 2 cryptochromes. Cryptochrome 1 had a more limited distribution, being found only among amphipods, branchiopods, copepods, decapods, euphausiids, and thecostracans (Fig. 4), while cryptochrome 2 was additionally found in isopods, stomatopods, and mysids (Fig. 5). However, care should be taken before making evolutionary or functional inferences, as this difference in distribution may reflect the tissue types included in the database. Nevertheless, the analysis shows the ease of application and potential for novel insights found in large-scale transcriptome analyses through the CrusTome database. Cryptochrome and photolyase protein sequences from CrusTome are available in Supplementary File 5.

### Future directions and applications

Biased taxon representation in public data repositories is a pressing issue for numerous fields within pancrustacean biology. CrusTome addresses this lack of taxonomic diversity by including fully assembled and preprocessed transcriptomes of underrepresented taxa. It is important to note that the taxonomic distribution of CrusTome's transcriptomes is ultimately dependent on sequence data that are publicly available and thus may be subject to biases. It is for that reason that CrusTome was envisioned as a community resource that will grow and evolve as data are produced and incorporated to continuously address taxonomic and tissue representation gaps. The authors also look forward to potential future collaborations with the developers of existing database solutions for crustaceans (i.e. CrusTF, CrustyBase, and CAT) to incorporate CrusTome into GUI-accessible resources. As the scope of the aforementioned projects differs from that of CrusTome, it is important to note that future work integrating these different databases, and leveraging the advantages of each, would be of great benefit to researchers using pancrustaceans as model systems.

### Conclusion

CrusTome provides a robust crustacean transcriptome database in easily accessible formats, using a consistent pipeline for increased reliability and comparability of results. A major goal is to provide a mechanism to improve the current paucity of accessible genomic and transcriptomic data for nonmodel crustaceans. This accessibility and ease of incorporation into existing pipelines enable analyses at larger scales. Moreover, CrusTome can be used to address long-standing questions in crustacean biology, such as molting and growth (Mykles and Chang 2020; Mykles 2021), sensory biology (e.g. vision and chemoreception; Pérez-Moreno *et al.* 2018; Kozma *et al.* 2020), convergent evolution (e.g. carcinization; Wolfe *et al.* 2021; Yang *et al.* 2021), or adaptation to extreme or changing environments (e.g. caves, deep-sea, and polar waters; Pérez-Moreno *et al.* 2016; DeLeo and Bracken-Grissom 2020; Andersen *et al.* 2022). It is our hope that the CrusTome database facilitates access to the rapidly growing number of genomes and transcriptomes being sequenced, particularly to those of nontraditional model organisms. As the transition into a posttranscriptomic era takes place, pancrustacean research must take full advantage of the large amounts of data produced by current and emerging technologies. A major aim of CrusTome is to bridge gaps of knowledge among pancrustaceans by including underrepresented taxa. Accessibility to the large amounts of raw data being deposited in public repositories enables scalable and integrative multiomic analyses that could ultimately lead to novel biological, ecological, and evolutionary insights across the tree of life (Mykles *et al.* 2010).

## Supplementary Material

jkad098_Supplementary_Data

## Data Availability

The CrusTome databases have been deposited in the Zenodo repository for public access under DOI: https://doi.org/10.5281/zenodo.7730440. Supplementary File 1 contains a spreadsheet with metadata regarding the raw data employed in building the CrusTome database. Among these data, accession IDs and sample identifiers are included. Additional links to the CrusTome database's associated metadata and example analyses (code, alignments, tree files, etc.) are available at the CrusTome GitHub site: https://github.com/invertome/crustome. Supplemental material available at G3 online.
