# Supplementary material for: CrusTome: a transcriptome database resource for large-scale analyses across Crustacea
Source: G3 (Bethesda). 2023 May 2;13(7):jkad098. doi: 10.1093/g3journal/jkad098 (PMC10320764; doi:10.1093/g3journal/jkad098)
Supplement: jkad098_Supplementary_Data [file jkad098_supplementary_data.zip › File_S4_G3-2023-404078.docx]

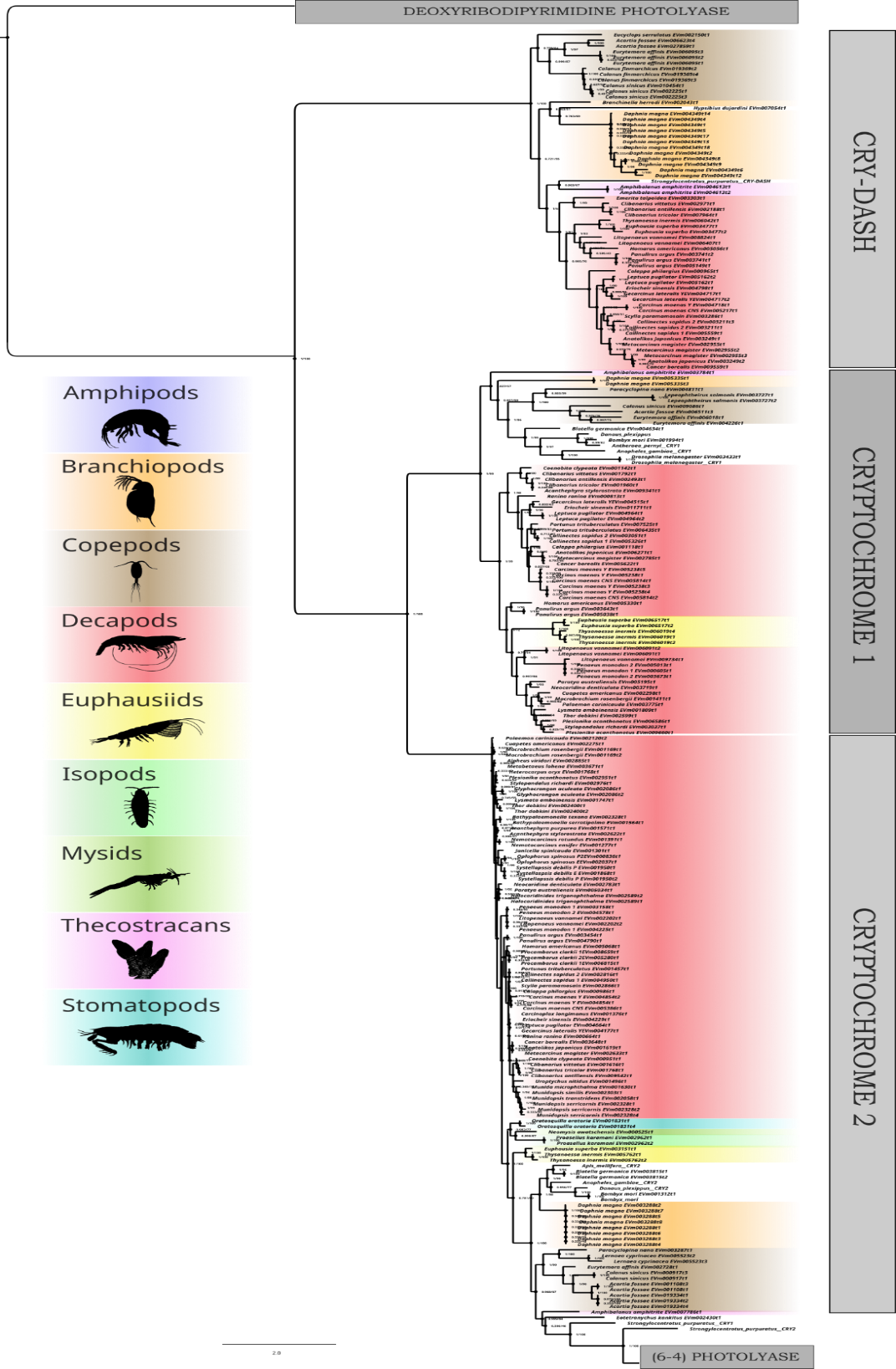
**S4**. Rooted phylogenetic tree (LG+R10) of Cryptochrome 1, Cryptochrome 2, Cryptochrome-DASH, found across transcriptomes of multiple crustacean species and tissues with the use of the CrusTome database. Representative taxa images from PhyloPic.org

**S4.** Rooted phylogenetic tree (LG+R10) of Cryptochrome-DASH found across transcriptomes of multiple crustacean species and tissues with the use of the CrusTome database. Representative taxa images from PhyloPic.org**
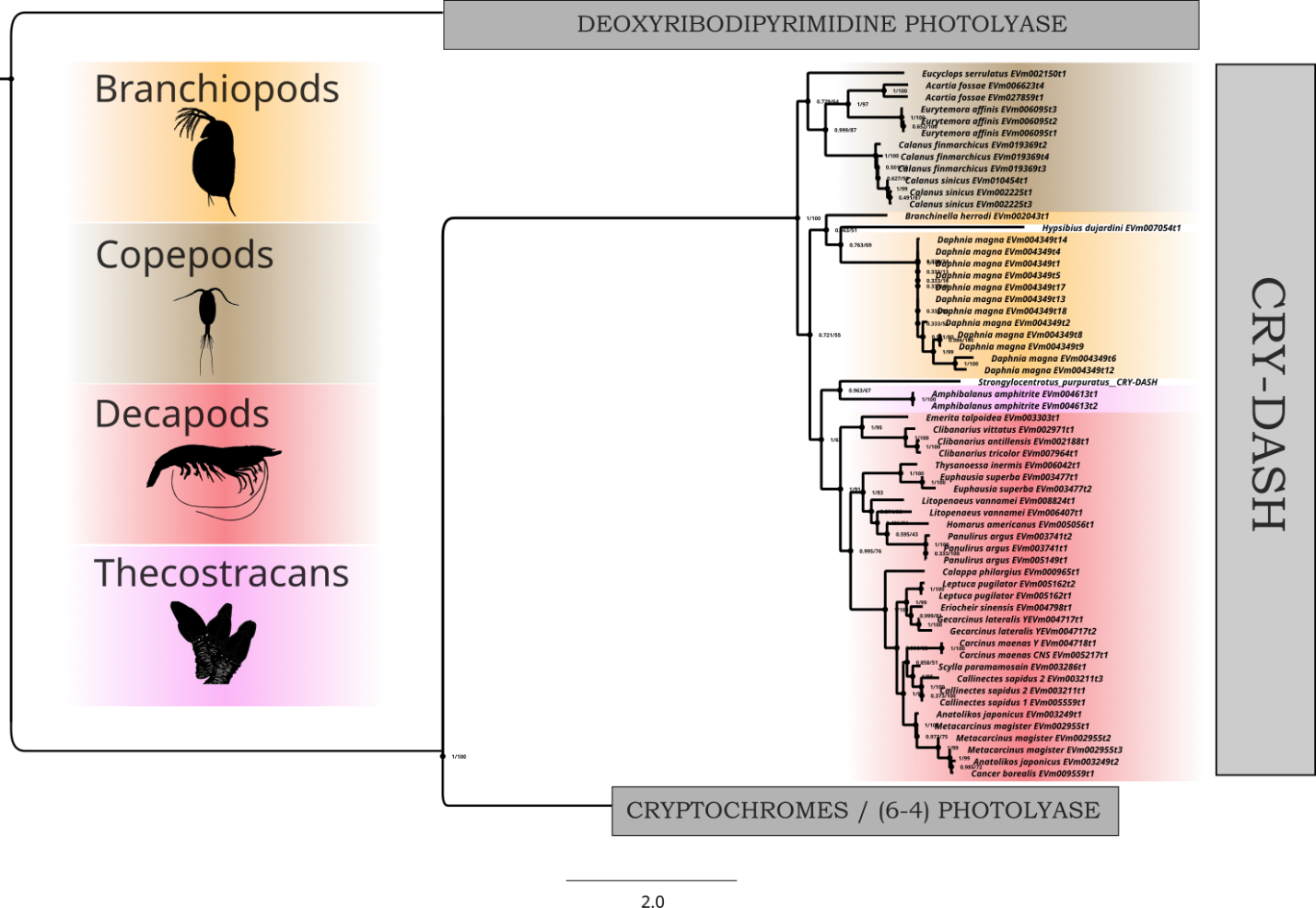
**

**S4.** Rooted phylogenetic tree (LG+R10) of D-Photolyase found across transcriptomes of multiple crustacean species and tissues with the use of the CrusTome database. Representative taxa images from PhyloPic.org**
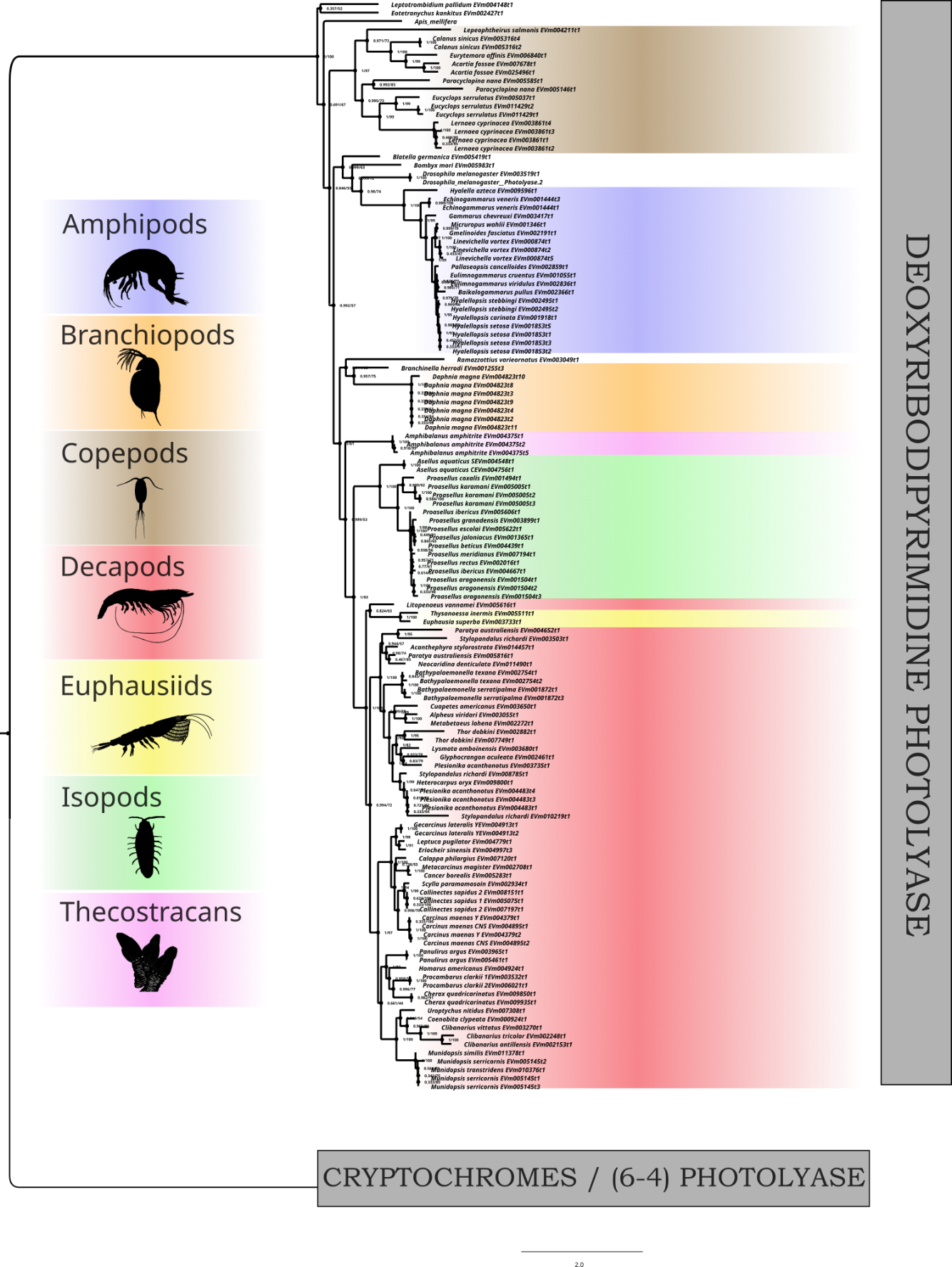
**
