# Supplementary material for: CrusTome: a transcriptome database resource for large-scale analyses across Crustacea
Source: G3 (Bethesda). 2023 May 2;13(7):jkad098. doi: 10.1093/g3journal/jkad098 (PMC10320764; doi:10.1093/g3journal/jkad098)
Supplement: jkad098_Supplementary_Data [file jkad098_supplementary_data.zip › Supplemental_Material_Legends_G3-2023-404078.docx]

**Supplementary Materials**

**File S1. *Metadata table. Attached as an Excel file, S1.SuppTable.xlsx***

**File S2. *Reference invertebrate cryptochrome and photolyase sequences. FASTA file, S2.cryptochromes.fasta***

**File S3*. Example code to conduct a phylogenetic analysis of pancrustacean cryptochromes and photolyases using the CrusTome database. Shell script, S3.cryptochromes.sh***

**File S4. *Additional phylograms of the CPF proteins. Attached as a Word document, S4.SuppFigs.docx***

**File S5. *CPF protein sequences found within CrusTome. FASTA file, S5.CPF.fasta***
